# Supplementary material for: Post-intervention epidemiology of STH in Bangladesh: Data to sustain the gains
Source: PLoS Negl Trop Dis. 2020 Dec 7;14(12):e0008597. doi: 10.1371/journal.pntd.0008597 (PMC7746288; doi:10.1371/journal.pntd.0008597)
Supplement: Attachment — Submitted filename: Response to Reviewers_Bangla_STH Epid 30 Sept 20.docx [file pntd.0008597.s001.docx]

**Authors’ Responses to Questions are in blue text below each reviewer question:**

**Methods**

- Are the objectives of the study clearly articulated with a clear testable hypothesis stated?

- Is the study design appropriate to address the stated objectives?

- Is the population clearly described and appropriate for the hypothesis being tested?

- Is the sample size sufficient to ensure adequate power to address the hypothesis being tested?

- Were correct statistical analysis used to support conclusions?

- Are there concerns about ethical or regulatory requirements being met?

**Reviewer #1: See below**

**Reviewer #2:**

- In various places (eg pages 9 and 12) it is stated that 12 Districts were surveyed and data from 10 of these are reported here.  I can't see the criteria for reporting a subset of data. If this is a partial view based on a decision of the authors this could result in significant bias.  A clear explanation is essential.
  - The first two districts (Bandarban and Nilphamari) were surveyed as a pilot to test the sampling methodology and gain field experience. Importantly, the survey teams could not access some of the randomly selected clusters due to local security issues which compromised sampling. Therefore, we decided to exclude these two surveys from the current analysis. This is clarified in line 34.
- It would be helpful to cite the sources of ethical clearance in the methods (they are attached to the file, but should be transparent to the reader of the paper too).
  - Bangladesh Medical Research Council reviewed and approved all surveys from an ethical perspective and also deemed this activity as “data for ongoing programme monitoring hence exempt from human subjects review”.
  - Under the Study design section (line 171-173), we have modified the text from, “Bangladesh Medical Research Council approved the survey protocol” to “Ethical clearance was obtained through the Bangladesh Medical Research Council (BRMC), who reviewed and approved the survey protocol.”

**Reviewer #3: (No Response)**

- Does the analysis presented match the analysis plan?

- Are the results clearly and completely presented?

- Are the figures (Tables, Images) of sufficient quality for clarity?

**Reviewer #1: See below**

**Reviewer #2:** **P13 line 80 states** "According to the 2011 national population census, this analysis represents about 15.5% of the Bangladesh population". It is unclear what this means. The sample is around 11,000 people, the population of Bangladesh is 160 million, so the sample is much less than 1%.

- Since each survey is powered to statistically represent results for that whole district population, the combined results from 10 districts are representative of 19,588,162 Bangladeshis living in those districts. However, the percentage should have been 14.1% of national population rather than 15.5%. We have corrected this number.

The enrolled sample is 17k and the specimens submitted are 11k, so almost 40% un-sampled.  Collecting stool specimens is notoriously difficult, but this seems a particularly low compliance. It is necessary to present an analysis of whether the compliance conceals bias: eg by gender, by age, locality etc. So that an assessment can be made of the consequences of the
40% under-sampling.

- We defined them as non-responsive with respect to providing a stool sample rather than un-sampled.
- The non-responders in our survey did participate in the interview process, however could not provide stool sample for any reason.
- Males (40.3%) were less likely to provide a stool specimen compared to their female (38.8%) counterparts (p-value 0.04).
  - It is likely that many males left early for their work and could not provide the stool samples.
- Significantly fewer PSAC (41.1%) and SAC (40.1%) provided stool specimens compared to adults (36.8%), p-value <0.001. We believe that adults may have a more regulated bowel routine while younger kids cannot be forced to produce it at a given time.
- To make it clear, we have added this to the manuscript, starting on line 187.

  **Reviewer #3: (No Response)**

**Conclusions:**
- Are the conclusions supported by the data presented?

- Are the limitations of analysis clearly described?

- Do the authors discuss how these data can be helpful to advance our understanding of the topic under study?

- Is public health relevance addressed?

**Reviewer #1: See below**

**Reviewer #2:**

**Page 17 line 130 states**: To our knowledge, this is the first time an impact evaluation of MDA directed at one specific risk group, SAC, has shown a significant reduction in STH prevalence across all age groups in a given community.  Suggest checking this reference, which shows this: Bundy, D.A.P., Wong, M.S., Lewis, L. and Horton, J. (1990) Control of geohelminths by delivery of targeted chemotherapy through schools.  Transactions of the Royal Society of Tropical Medicine and Hygiene 84, 115-120.

- Thank you very much for sharing this reference. We have modified the discussion section indicating the referenced article that showed decreased prevalence among other age groups while the intervention was primarily aimed at the 2-15 year old school going children.

**Page 19 line 176**: This is a rather limited list of possible explanations for variation in outcomes across districts.  I am particularly surprised to see no mention of two factors known to be important: WASH coverage and economics.  At present, the discussion is speculative and uninformative.  The authors should make this point quantitatively. Data are available for these
10 districts, which would allow comparison/ranking using government and/or World Bank data on both WASH and the economy.

- Since the main purpose of this study was to evaluate the impact of MDA on STH prevalence at the community level, we did not look at the economic and WASH factors and their impact on STH prevalence.
- We are using national level data below as district-specific socio-economic data and development indices are not available to the authors at this time and were not part of the original study question and design. This, of course, does not preclude further detailed investigations along these areas once the district level STH epidemiologic results are made available to all.
- According to JMP data shared by WHO and UNICEF, Bangladesh has made significant improvement in providing access to improved latrines from 37.9% (2008) to 52.2% (2017) of total population.
- World bank data shows that:
  - the Gross National Income (GNI) per capita has increased more than 165% from USD 660 (2008) to USD 1750 (2018)^[1]^
  - infant mortality rate has gone down from 43.2 (2008) to 25.1 (2018) per 1,000 live births
  - the prevalence of undernourishment has been declining, life expectancy is continuously increasing and the literacy rate is also going up.
- We have added text on this from line 299 onwards.

**Page 20 194-196**: the point about the transition from MDA to a mixture of self-purchasing in better off areas and MDA in poorer is important. There should be specific reference here to the work of Nilanthi Da Silva on exactly this topic in Sri Lanka, where public MDA only now persists in the poorest "plantation" areas.  If the authors here rank districts by economics (the point above) then they could make this call specifically.

- We have already cited the referenced article in our discussion (please see line 327).

**Reviewer #3: (No Response)**

- Editorial and Data Presentation Modifications?

Use this section for editorial suggestions as well as relatively minor modifications of existing data that would enhance clarity. If the only modifications needed are minor and/or editorial, you may wish to recommend “Minor Revision” or “Accept”.

**Reviewer #1: See below**

**Reviewer #2: NA**

**Reviewer #3: (No Response)**
**Summary and General Comments**:

Use this section to provide overall comments, discuss strengths/weaknesses of the study, novelty, significance, general execution and scholarship. You may also include additional comments for the author, including concerns about dual publication, research ethics, or publication ethics. If requesting major revision, please articulate the new experiments that are needed.

**Reviewer #1**: General: Dhakal and co-authors present a well-written account of a rigorous survey on soil-transmitted helminth (STH) prevalence and selected risk factors across Bangladesh. Such high-quality studies are invaluable for program management, and are examples for other programs facing the question whether to continue interventions or adapt to changing conditions.
**A few comments are offered for clarification and discussion**:

1) **Line 45**: It is unclear how the survey districts were selected
   a) The Bangladesh national programme (ELFSTH) selected all the survey districts to generally represent the ecological zones, urban/rural areas etc. according to programmatic priorities (updated Fig. 6).
2) **Lines 50-51**: Supposedly, the word “years old” is missing after the figure
   a) Thank you very much for pointing it out. It was an inadvertent error that is now corrected.
3) **Line 70**: Provide an estimate on the time lapse between sample production and sample analysis (e.g. “analyzed on the day of sample collection”)
   a) Stool collection receptacles were handed after the interview and the samples were collected the following morning (7:00 am to 9:00 am). All samples were transported to the laboratory in a cooler box within three hours of collection; within an average of 10 hours (see line 152-153).
4) **Line 85**: Was the survey really powered to detect a certain prevalence of MHII?
   a) The survey was powered to detect the threshold of STH prevalence (and MHII) ≥10%.
5) **Line 94**: The figure given in the abstract and here on the number of individuals submitting stool samples is not identical
   a) We would like to clarify that in total 11,022 stool samples were collected and 198 were excluded during the data cleaning process for several reasons as highlighted in line 176 onwards. This “data cleaning” process led to the final number of 10,824 stool samples which were used for the analyses. Therefore, our results are based on the final 10,824 stool samples.
   b) Thank you very much. To clarify these discrepancies, we have modified the text from
      i) “10,824 (61%) provided stool samples” to “our results are based on 10,824 (61.0%) stool samples” (page 2, line 38).
6) **Line 99**: Basic data on the ~40% who did not provide samples should be provided (variation between districts, age class and sex distribution)
   a) Please find the requested gender and age breakdown from line 187 on. There were no significant differences across districts across these categories.
7) **Line 104**: Ascaris, Trichuris: please write the proper species name/abbreviation
   a) Thank you very much. We have corrected this.
8) **Line 131**: The data are intriguing but there is no evidence for causality between school-based MDA and the prevalence reduction. Some points are discussed below, and the authors should be careful to not over-interpret their data or suggest causality.
   a) Thank you for this observation. The survey was not designed to elucidate the causal relationship between MDAs and the prevalence. On the basis of our data, we cannot say there is an actual cause and effect relationship between school-based MDA and the reduction in STH prevalence. However, since high coverage rates were reached consistently for more than 10 years in Bangladesh, we can say that this was likely a major factor in the observed decrease of STH prevalence and intensity, (even though baseline prevalence is estimated from limited data derived from methods different than our own).
9) Are there any plans to sample districts that were not covered in this survey?
   a) There may be additional surveys conducted under the leadership of the Bangladesh ELFSTH Programme and dictated by program needs and available resources.
   b) This is a decision for the ELFSTH Programme and influenced by many factors outside the scope of this manuscript.
10) Is there any information on the timing of sample collection compared to the last deworming event?
    a) The gap between the survey and the previous deworming event was at least five (5) months (see line 132)
11) Some abbreviations are used without introduction, or they are introduced after their first use (e.g. ICSPM, ELFSTH)

- Thank you very much for identifying these language errors. We have corrected them in the updated manuscript.

**Reviewer #2**: This paper explores an important and topical issue. The finding that treating school children only has an impact on transmission as a whole was made some 40 years ago by R. M. Anderson, but has been demonstrated only rarely (but it has been demonstrated before - see reference in comments above).  There is a current trend in the literature to argue for treatment of all ages as essential to significant interruption of transmission to significantly lower levels.  This paper is one of a very few to show that this is not necessary.  This is such an important and topical point that I am surprised that the authors do not make more of it.  School based treatment is so much less costly and easier, which is why the programme has been self-sustainable in Bangladesh.

- Thank you for highlighting the policy implications and importance of our paper. While we do observe the very important incidental finding (not planned in the original study design) of community-driven, self-deworming that **may** be a result of the school-deworming, we really do not have the data to make that causal association. We agree that this is a very important finding towards sustainability discussions and policies, etc. which is why we recommend strongly that further socio-behavioral studies be conducted to clearly identify the factors behind this observed behavior. Of note: our study had a much larger sample size than the original study by Prof. Bundy et al, so provides better statistical support to their earlier observation. Some other studies and modeling have not shown the same correlation between school treatments and community-wide effects (especially in Sub Saharan Africa, probably because of the hookworm prevalence, mainly an adult infection, being higher in those countries).
- Based on findings from ICSPM surveys we cannot say the ELFSTH program in Bangladesh is self-sustaining. The program primarily relies on drugs donated by pharmaceutical partners through WHO with program implementation costs defrayed by developmental partners.

On a related point, a very novel finding of this study is the very high rates of adult and PSAC self-treatment using out-of-pocket funds.  This supports the contention that school based public treatment will be supported by out-of-pocket self treatment by those who can afford to do so.  This was the experience in Sri Lanka that led to their being able to refine, reduce and target their school based MDA programme.  These are important policy implications that deserve stronger mention.

In describing the outcomes the authors use the figure of 79.8% as the baseline figure from 2005, based on a government report.  The details of this baseline survey need to be described here. Was this for the same 10 Districts as here; was it the same age groups; same Dx? etc

- The authors need to be able to make the case that the comparison with the present result
is valid.  If it is not, then the very low prevalence seen today still stands as a cross country sampling of the status of infection after treatment, but clearly needs to be presented in that way.

- Please see the above response to behavioral observation on self-treatment. The community-level behavioral changes are, indeed, very promising and noteworthy and are mentioned as such in the results and discussion. However, the surveys were not designed to elicit a causal relationship between these “incidental” findings and school-based deworming. We have advocated for the need to address this causal association and related factors through follow up behavioral studies. Understanding these factors, in addition to current disease epidemiology, would be of tremendous value towards sustaining the impact of school deworming with national resources.
- The 2005 study was conducted in five schools each from Feni, Khagrachari, and Patukali districts with support from WHO. The detailed data from the 2005 surveys are not available.
- There were additional STH surveys conducted after 2005 but the methods, sampling and quality of data were not documented. Comparatively, the 2005 survey methods were better defined so we used those results as the baseline.

**Reviewer #3**: **Comments and observations**
Overall, the manuscript is relevant, well written and with study design according to WHO recommendations. It provides useful information regarding the impact of more than 10 years mass drug administration interesting on prevalence and intensity of STH infections in Bangladesh. Nevertheless, the manuscript needs to be improved regarding some aspects of methodology, results, and discussion.

**Methodology:**

1. It should be important to describe whether the different districts and communities have comparable characteristics or are in different settings: ecological settings or variation, vegetation, climate, seasonality and water contact activities and behavior.
   a. We have added an additional map (Fig. 6) showing the ecology, vegetation, and climate of selected districts and text in the discussion (line 291-294).
2. Describe the main occupation of the population and their likely exposure to STH transmission.
   a. We have clarified the most common occupations of survey participants by adding: “The three most commonly reported occupation were students (34.6%) housewives (25.6%), other (21.8%)” (line 185-186).
3. If possible, the authors should give some information on health facilities in relation to STH: diagnostic facilities, availability of praziquantel.
   a. Praziquantel is not part of the STH control strategy and was not used by the ELFSTH programme in the study districts. Bangladesh does not have a schistosomiasis treatment programme (line 365-366).
4. Provide short information regarding WASH activities, hygiene, and sanitation.
   a. We are currently exploring the relationship of observed STH prevalence with WASH variables collected during the survey. Once the analysis is completed, we plan to submit it in another manuscript separately.
5. It is not clear whether the treatment has been provided to the children following the examination.
   a. ELFSTH national program office communicated all the positive results to survey participants through the district health officers for further management and treatment.

**Results**:

1. The authors should provide additional Table(s) showing the results of prevalence and if possible, the intensity of STH infections in 2005 compared to 2017-2020 in different district to justify the objective of the study.
   a. As responded earlier, we do not have access to the 2005 survey data and so are unable to further analyze, compare or produce additional tables.

**Discussion**:

1. Results of differences in prevalence and intensity need to be more explained.
   a. Why the highest STH prevalence in Sunamganj, Bhola and Sirjganj in contrast to others?
      i. Thanks for raising this important question. This was the main reason for conducting these surveys so the ELFSTH program could use its scarce resources on areas and populations with persistent high infection transmission. CWW and ELFSTH are conducting further assessments of these “hot spots” using standard tools and assessment approaches which will be submitted as a separate manuscript. Early investigation points to service area challenges (low coverage due to multiple programmatic and local challenges) and community misconceptions as well as health communication issues.
   b. The same question goes for the intensity.
      i. Same response as above.
2. What do specifically, the authors mean by ecological variation?
   a. ELFSTH program has divided the country into four main ecological categories based upon terrain, climate and dry/wet categories: coastal (next to the Bay of Bengal), flood prone (low-lying areas regularly flooded with major rivers), plane (fertile land) and low hills. We added a map (Fig. 6) showing these categories for the surveyed districts.

**Recommendation**
The manuscript could be accepted after considering the comments and
observations with “major changes”

--------------------

PLOS authors have the option to publish the peer review history of their
article ("<https://journals.plos.org/plosntds/s/editorial-and-peer-review-process#loc-peer-review-history>"

- What does this mean?
  - If published, this will include your full peer review and any attached files.
  - If you choose “no”, your identity will remain anonymous but your review may still be made public.
  - Do you want your identity to be public for this peer review?
  - For information about this choice, including consent withdrawal, please see our
    "<https://www.plos.org/privacy-policy>"

**Reviewer #1**: Yes: Peter Steinmann

**Reviewer #2:** No

**Reviewer #3**: Yes: Moussa Sacko, PhD


**Figure Files:**

While revising your submission, please upload your figure files to the Preflight Analysis and Conversion Engine (PACE) digital diagnostic tool, [https://pacev2.apexcovantage.com](https://pacev2.apexcovantage.com/). PACE helps ensure that figures meet PLOS requirements. To use PACE, you must first register as a user. Then, login and navigate to the UPLOAD tab, where you will find detailed instructions on how to use the tool. If you encounter any issues or have any questions when using PACE, please email us at [figures@plos.org](mailto:figures@plos.org).

**Data Requirements:**

Please note that, as a condition of publication, PLOS' data policy requires that you make available all data used to draw the conclusions outlined in your manuscript. Data must be deposited in an appropriate repository, included within the body of the manuscript, or uploaded as supporting information. This includes all numerical values that were used to generate graphs, histograms etc. For an example see here:
<http://www.plosbiology.org/article/info%3Adoi%2F10.1371%2Fjournal.pbio.1001908#s5>.

**Reproducibility**:
To enhance the reproducibility of your results, PLOS recommends that you deposit laboratory protocols in [protocols.io](http://protocols.io/), where a protocol can be assigned its own identifier (DOI) such that it can be cited independently in the future. For instructions see <http://journals.plos.org/plosntds/s/submission-guidelines#loc-materials-and-methods>

__________________________________________________
In compliance with data protection regulations, you may request that we remove your personal registration details at any time.  (Use the following <https://www.editorialmanager.com/pntd/login.asp?a=r>). Please contact the publication office if you have any questions.

1. https://data.worldbank.org/country/bangladesh?view=chart
